# Universal Dermal Microbiome in Human Skin

**DOI:** 10.1128/mBio.02945-19

**Published:** 2020-02-11

**Authors:** Lene Bay, Christopher James Barnes, Blaine Gabriel Fritz, Jonathan Thorsen, Marlene Elise Møller Restrup, Linett Rasmussen, Johan Kløvgaard Sørensen, Anne Brun Hesselvig, Anders Odgaard, Anders Johannes Hansen, Thomas Bjarnsholt

**Affiliations:** a Costerton Biofilm Center, Department of Immunology and Microbiology, University of Copenhagen, Copenhagen, Denmark; b Centre for GeoGenetics, Natural History Museum, University of Copenhagen, Copenhagen, Denmark; c Copenhagen Prospective Studies on Asthma in Childhood, Herlev-Gentofte Hospital, University of Copenhagen, Copenhagen, Denmark; d Orthopaedic Department, Herlev-Gentofte Hospital, Gentofte, Denmark; e Department of Clinical Microbiology, Rigshospitalet, University of Copenhagen, Copenhagen, Denmark; University of Pittsburgh School of Medicine

**Keywords:** 16S rRNA genes, cutaneous compartments, DNA sequencing, dermal microbiota, dry habitat, skin biopsies, skin microbiome

## Abstract

Human skin microbiota has been described as a “microbial fingerprint” due to observed differences between individuals. Current understanding of the cutaneous microbiota is based on sampling the outermost layers of the epidermis, while the microbiota in the remaining skin layers has not yet been fully characterized. Environmental conditions can vary drastically between the cutaneous compartments and give rise to unique communities. We demonstrate that the dermal microbiota is surprisingly similar among individuals and contains a specific subset of the epidermal microbiota. Variability in bacterial community composition decreased significantly from the epidermal to the dermal compartment but was similar among anatomic locations (hip and knee). The composition of the epidermal microbiota was more strongly affected by environmental factors than that of the dermal community. These results indicate a well-conserved dermal community that is functionally distinct from the epidermal community, challenging the current dogma. Future studies in cutaneous disorders and chronic infections may benefit by focusing on the dermal microbiota as a persistent microbial community.

## Abstract (precis)

Human skin microbiota is thought to be unique according to the individual's lifestyle and genetic predisposition. This is true for the epidermal microbiota, while our findings demonstrate that the dermal microbiota is universal between healthy individuals. The preserved dermal microbial community is compositionally unique and functionally distinct to the specific environment in the depth of human skin. It is expected to have direct contact with the immune response of the human host, and research in the communication between host and microbiota should be targeted to this cutaneous compartment. This novel insight into specific microbial adaptation can be used advantageously in the research of chronic disorders and infections of the skin. It can enlighten the alteration between health and disease to the benefit of patients suffering from long-lasting socioeconomic illnesses.

## INTRODUCTION

Human skin contains highly individual epidermal microbiota (1–7), which is a diverse and complex community (3). This community exhibits temporal changes (4), adapts to its surroundings (1), and is shaped by chemical, biological, and physical conditions on the skin (1, 8, 9). Community composition varies systematically among different skin habitats, such as between dry, moist, and sebaceous skin (1, 3, 4, 9, 10). This may partially be driven by the density of glands and hair follicles (11–13). Large bacterial aggregates have recently been observed deep within hair follicles (14), but these communities can be sampled only by skin biopsies. Most studies examining human skin microbiota utilize cotton swabs (1, 3–5, 8, 10, 15) and, therefore, collect only the epidermal microbiota (9). Few investigations have examined the distribution of the microbiota in full-thickness skin biopsy specimens (9) or within subepidermal compartments (2) A complete characterization of the composition and distribution of the entire skin microbiota (1) within various cutaneous compartments and structures is essential in order to understand the role of microorganisms within the human skin. This study investigates the effect of skin depth and anatomic location on bacterial composition in healthy skin microbiota. Full-thickness skin biopsy specimens were collected at two different anatomic locations with a sample size exceeding that of previous human studies (1–4, 9, 16). The biopsy specimens were separated along the boundary of the epidermis and dermis. High-throughput sequencing (HTS) of the 16S rRNA bacterial region was performed to comprehensively analyze the microbial communities, extracted directly from their natural environment.

## RESULTS

### Microbial composition and richness.

**FIG 1:**
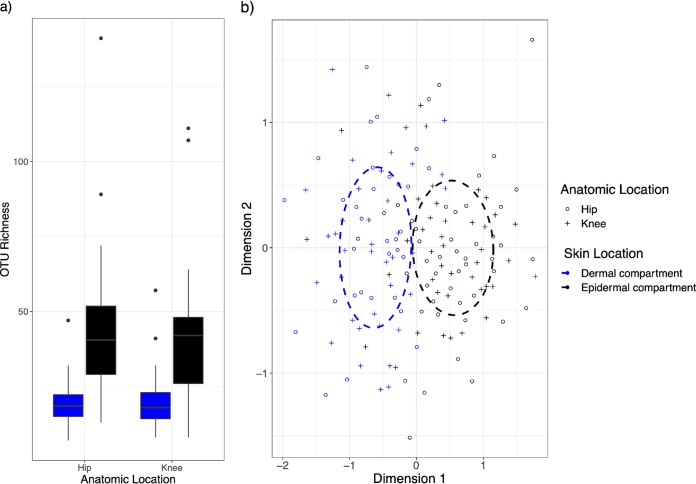
Bacterial composition in box plot and NMDS plot. (a) OTU richness by anatomic location (hip versus knee) and skin compartment (epidermal versus dermal). There were no differences in OTU richness between hip and knee. OTU richness increased from the dermal to the epidermal compartment. (b) An NMDS plot of bacterial 16S composition with clear grouping by skin location. Skin compartment and anatomic location are represented by point color and shape, respectively, while dotted lines represent a 95% reference interval for the skin compartment.

Skin compartment (dermal and epidermal) significantly affected the composition of operational taxonomic units (OTU) (*P* = 0.001, df = 1) and OTU richness (*P* < 2e−16, df = 4) but was not affected by anatomic location (hip and knee) (*P* = nonsignificant [ns]) (Fig. 1a). This skin depth effect was evident in the nonmetric multidimensional scaling (nMDS) plot, where dermal and epidermal samples grouped separately and the majority of samples clustered within the 95% confidence intervals (Fig. 1b).

### Similarities within compartments.

Given the divergent bacterial communities between the epidermal and dermal compartments, these communities were separated before their community variation and regulation were analyzed independently. Median similarity between samples was calculated using Jaccard similarity matrices, being 0.256 and 0.286 in the epidermal and dermal compartments, respectively (see Fig. S2 in the supplemental material), with the dermal community being significantly more similar and conserved (*W* [Wilcoxon rank sum] = 5,328,400, *P* < 0.001) than the more variable epidermal community.

10.1128/mBio.02945-19.3 FIG S2 Similarity within cutaneous compartments. The graph shows the values for density versus the median Jaccard similarity between samples within the epidermal and dermal compartments, which were 0.258 and 0.286, respectively. A significantly higher similarity (*P* < 0.001) was observed within the dermal compartment. Download FIG S2, PDF file, 0.02 MB. Copyright © 2020 Bay et al. 2020 Bay et al. This content is distributed under the terms of the Creative Commons Attribution 4.0 International license.

### Correlation to metavariables.

Three individual analyses, envfit, mixed linear modeling, and multivariate general linear modeling, tested variations in the epidermal and dermal communities against metadata variables (age, sex, smoking, diabetic status, and interpersonal variation). Mixed linear modeling was performed to test the metadata variables that drive variation of bacterial richness in the epidermal compartment, finding that both age (*P* < 0.001) and diabetic status (*P* = 0.007) had significant effects (Table S1). Meanwhile, envfit and multivariate general linear modeling were performed to test whether metadata parameters drive variation in the epidermal compartment bacterial composition, with both showing that age (*P* = 0.002 and 0.006, respectively), smoking habits (*P* = 0.011 and 0.020, respectively), and interpersonal variation (*P* = 0.001 and 0.002, respectively) drive epidermal compartment variation, while multivariate general linear modeling also found compositional variation associated with anatomic location (*P* = 0.046) and sex (*P* = 0.020). Controversially, no metadata parameters were shown to affect bacterial richness in the dermal compartment when mixed linear modeling was used, while the dermal compartment’s bacterial composition was shown to vary with only age (*P* = 0.010) and interpersonal variation (*P* = 0.004) when multivariate general linear modeling (Table S1) was used and with no parameters when envfit was used.

10.1128/mBio.02945-19.9 TABLE S1 Overview of the statistics listing the *P* values from the tests used. Significant *P* values are highlighted in bold. n/a, not available. Download Table S1, DOCX file, 0.01 MB. Copyright © 2020 Bay et al. 2020 Bay et al. This content is distributed under the terms of the Creative Commons Attribution 4.0 International license.

### Taxonomy in dermal and epidermal compartments.

**FIG 2:**
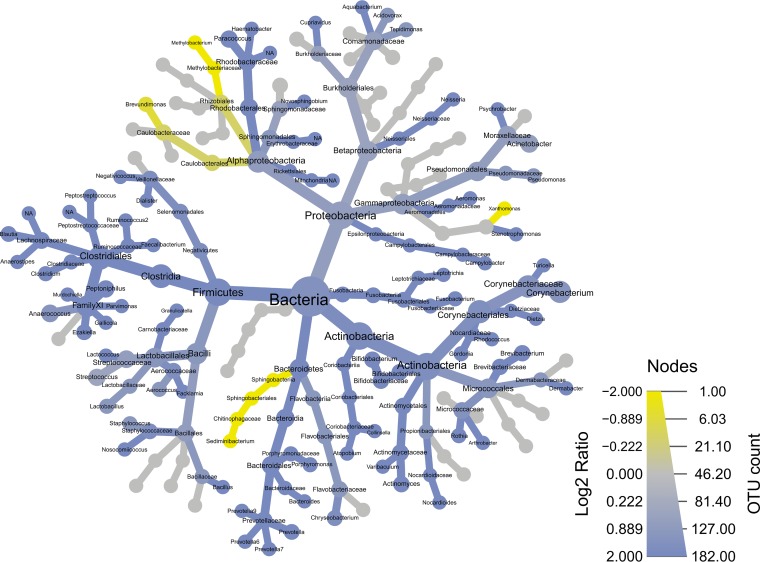
Heat tree illustrating the overall taxonomy of the bacterial community across the dermal compartment relative to that of the epidermal compartment (see the supplemental information). Color changes represent the difference in log_2_ ratio of median proportions of reads between epidermal and dermal compartments. The blue nodes are more enriched in the epidermal compartment, while yellow nodes are more enriched in the dermal compartment. The gray nodes are equally present in both compartments.

The taxonomy of dermal and epidermal compartments is illustrated by a heat tree (Fig. 2). Nodes represent taxonomic levels, and each branch represents the relationship between those entities. OTU richness is represented by node size, color, and color intensity, where blue branches are more present in the epidermal compartment and yellow branches are more represented in the dermal compartment. Gray branches appear equally in both compartments. This heat tree emphasizes that the differences across skin layers are driven by increased bacterial diversity in the epidermal compartment.

Other than the phylum *Proteobacteria*, which showed a significantly higher (*P* < 0.001) estimated mean relative abundance (%) and an similar estimated mean log OTU richness, the top four most persistent phyla were all significantly less rich (*P* < 0.001), with significantly lower mean relative abundances (*P* < 0.001) in the dermal compartment than in the epidermal compartment (Fig. S5a).

10.1128/mBio.02945-19.6 FIG S5 Box plot of top four phyla and top five genera in dermal and epidermal compartments. The estimated mean log (OTU richness) and the estimated mean relative abundance (%) indicate differences between the dermal and epidermal compartments. Download FIG S5, PDF file, 0.4 MB. Copyright © 2020 Bay et al. 2020 Bay et al. This content is distributed under the terms of the Creative Commons Attribution 4.0 International license.

The estimated mean log OTU richness of the top five most persistent genera was similar between compartments, except that for *Corynebacterium* spp., which was significantly higher (*P* < 0.001) in the epidermal compartment. Correspondingly, the estimated mean relative abundance (%) of *Corynebacterium* spp. was significantly higher (*P* < 0.001) in the epidermal compartment, while that of *Pelomonas* spp. was significantly higher (*P* < 0.001) in the dermal compartment. The remaining most persistent genera were similar in relative abundance between compartments (Fig. S5b).

### Indicator species analysis.

**FIG 3:**
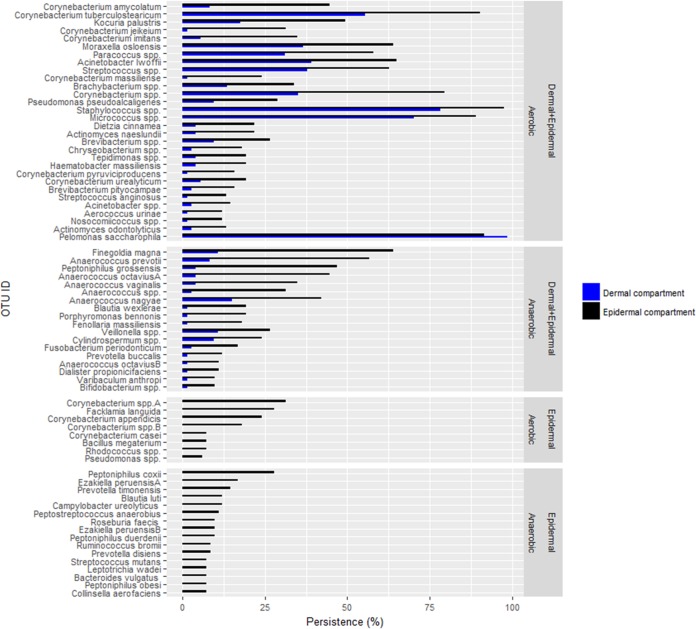
Persistence plot. The persistence of 75 genera/species that differ significantly in persistence between the dermal and epidermal compartments. They are grouped according to their anaerobic or aerobic preference and their presence in both the epidermal and dermal compartments or in the epidermal compartment only. Of the genera/species significantly different in persistence between the two compartments, 61% were aerobic and 39% were anaerobic. Among the genera/species present only in the epidermal compartment, 35% were aerobic and 65% were anaerobic.

OTUs that significantly differed in persistence between the two cutaneous compartments (Sidak’s alpha < 0.05) were identified using indicator species analysis and plotted in a persistence plot (Fig. 3). Seventy-five OTUs differed significantly, and these were grouped into aerobic or anaerobic species (based on published literature) that were significantly persistent in epidermal and dermal compartments. All OTUs were more persistent in the epidermal compartment with the exception of a single Pelomonas saccharophila OTU.

### Prediction of genes and pathways.

**FIG 4:**
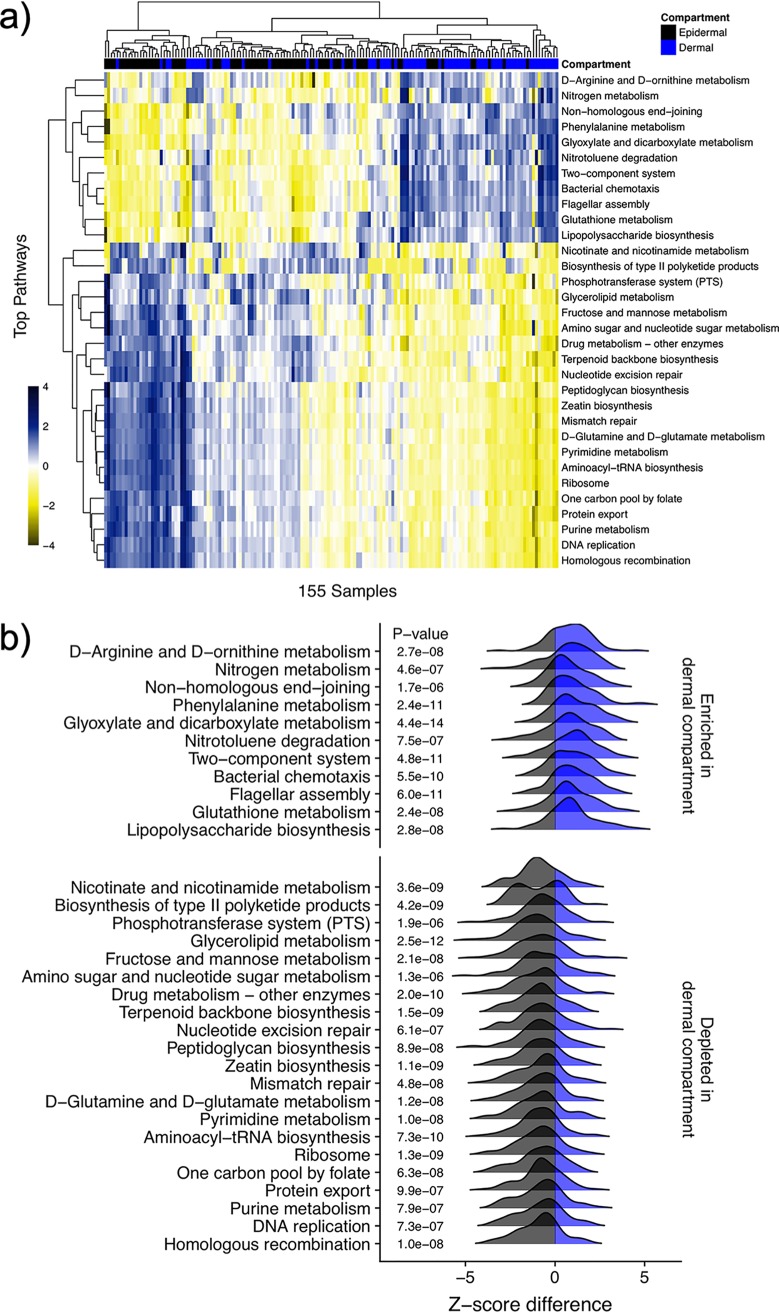
Heat map and ridge plot of predicted pathways. Predicted relative abundances of the pathways that differ the most between dermal (blue) and epidermal (black) compartments (all *q* values < 0.05). (a) (a) Heat map of log Z-scores of relative abundances visualized in blue (enriched) to yellow (depleted); (b) kernel density plots of differences between dermal and epidermal compartments from all paired samples. Trees are built with hierarchical clustering.

A principal-component analysis (PCA) plot of the predicted genes demonstrates separate groupings of samples from the dermal and epidermal compartments (Fig. S6) based on predicted gene ortholog abundances. There was a significant difference (*P* < 1e−5) in relative gene abundance between compartments, suggesting a difference in overall functionality of the microbial communities. A heat map (Fig. 4a) and a simplified ridge plot (Fig. 4b) describing the 25 most significantly different pathways between compartments clearly illustrate the variability in functionality throughout the stratified tissues. See the supplemental material for a more detailed heat map of all significantly different pathways between compartments (Fig. S7).

10.1128/mBio.02945-19.7 FIG S6 Principal-component analysis of predicted relative gene abundances. Each dot represents a sample, and a systematic shift between the dermal and epidermal compartments is noted (*P* < 1e−5). Values were log Z-scored prior to computation. Download FIG S6, PDF file, 0.01 MB. Copyright © 2020 Bay et al. 2020 Bay et al. This content is distributed under the terms of the Creative Commons Attribution 4.0 International license.

10.1128/mBio.02945-19.8 FIG S7 Heat map of all predicted relative pathway abundances, which are significantly different between the dermal and epidermal compartments (*q* < 0.05). Individual pathways are shown as log Z-scores of relative abundances. Trees are built with hierarchical clustering. Download FIG S7, PDF file, 0.1 MB. Copyright © 2020 Bay et al. 2020 Bay et al. This content is distributed under the terms of the Creative Commons Attribution 4.0 International license.

## DISCUSSION

Different anatomic locations were expected to have different compositions and richness (4), but we demonstrated that neither bacterial community composition nor overall bacterial richness (as operational taxonomic units [OTU]) differed between the anatomic locations (Fig. 1a). This challenges previous findings, in which the cutaneous community composition differed greatly between skin locations, even within habitats (4). While local conditions, such as density of glands or hair follicles, as well as chemical factors, such as pH, moisture, and temperature, all influence bacterial composition (1, 4, 12, 15), the hip and knee are both dry skin habitats with similar topographical conditions, which may explain why no significant differences were observed. Additionally, while a strong and significant patient effect was expected based on many previous studies (1, 3–7, 17), this was limited to the epidermal communities, and no effect on the dermal communities was detected at all (for skin compartments: see Fig. S1a and b in the supplemental material).

Differences in skin compartments had the largest effect on determining the overall community composition. The dermal community consisted of a specific subset of the epidermal community, but the stark contrast between dermal and epidermal bacterial communities was surprising. Previous attempts to analyze divided skin biopsy specimens by 16S rRNA sequencing found similar microbial profiles in both compartments (9), whereas in our study, bacterial community composition and richness (as OTU richness) differed significantly between the dermal and epidermal compartments (Fig. 1). Additionally, when comparing community similarities (using Bray-Curtis similarity matrices), we found that the dermal compartment’s community was significantly less variable than that of the epidermal compartment (Fig. S2). The heat tree (Fig. 2) depicts the difference in OTU richness associated with each phylogenetic group and illustrates that the majority of OTUs were enriched in the epidermal compartment (blue), while relatively few groups demonstrated higher richness in the dermal compartment (yellow). The results here suggest that the bacterial community of the dermal compartment is less rich across nearly all taxonomic groups.

The distributions of the four major phyla were similar to those of previous investigations in human skin (1, 4, 9, 18). Other than the more abundant *Pelomonas* spp. within the phylum *Proteobacteria*, the most persistent genera showed abundance in the dermal community that was similar to or lower than the abundance in the epidermal community. Proteobacteria are found to colonize deep, cutaneous compartments and are presumably involved in regulating skin homeostasis between the host and the environment (19). Specifically, the dermal highly abundant *Pelomonas* spp. are one of the core commensals in cutaneous communities (19). Concurrently, *Corynebacterium*, a genus that is predominant in moist skin habitats but also prevalent in dry skin habitats (12), was mainly abundant and richer in the epidermal compartment than in the dermal compartment (Fig. S5). Together, these results suggest that the dermis, with its less complex and less variable bacterial community, is more specifically colonized (2).

To further characterize the differences between skin compartments, we analyzed OTUs that significantly differed in persistence between dermal compartments. An OTU may have a low abundance within the microbiome but nevertheless may be observed in many samples, suggesting a functional role. We define persistence as the percentage of samples in which an OTU is found (Fig. 4). A total of 75 OTUs differed significantly in persistence between the epidermal and dermal compartments. These OTUs were categorized as being present in both compartments or only the epidermal compartment and were classified into anaerobic or aerobic bacteria based on published literature findings. All OTUs, except Pelomonas saccharophila, were more persistent in the epidermal compartment, while there were no clear trends in the distributions of anaerobic and aerobic bacteria between the skin layers. Similarly, only a few individual OTUs increased in abundance within the dermis, which included a *Methylobacterium* species and a *Brevundimonas* species. These have been recorded as opportunistic pathogens in immunocompromised patients or in those suffering from an underlying disease (20, 21), and the dermis may serve as a reservoir for potential pathogens for patients at risk of skin and soft tissue infections. This is in agreement with a growing body of literature suggesting that a lack of regulation of the microbiome by the host (dysbiosis) can lead to previously commensal bacterial species becoming pathogenic (22). However, it should be noted that *Methylobacterium* spp. and other genera such as *Xanthomonas* spp. have previously been identified as common contaminants in DNA extraction kits (23). In our study, these genera were absent in our negative controls and were more abundant in the dermal communities, suggesting a role in the local microbiota rather than a systemic contamination, while other OTUs assigned to *Atopobium*, *Cellulomonas*, and *Conchiformibius* genera were likely contaminants.

Given that the dermal and epidermal bacterial communities differed in composition and richness, we examined the factors that regulate these differences. The effects of age, sex, smoking status, diabetic status, anatomic location, and interpersonal variation on the bacterial OTU richness of each skin compartment were modeled using generalized linear modeling, while envfit and multivariate general linear modeling (Table S1) were used to estimate their effects on the bacterial community composition. There was evidence that age, smoking status, diabetic status, and interpersonal variation affected the epidermal composition and bacterial OTU richness. There was also additional support that sex and anatomic location affected composition but not OTU richness (Table S1 and Fig. S3 and S4). The dermal microbiota was considerably less affected by these factors, with only interpersonal variation and age affecting bacterial composition, while no factor correlated with the dermal bacterial OTU richness. The results here therefore suggest that the epidermal bacterial composition was more affected by external factors, resulting in its higher variability. This is in accordance with the previous findings of many studies, where significant interpersonal variability was observed (6, 12, 16, 24–28). This variation is likely to be driven by greater exposure of the epidermis to the external environment. It is also quite possible that compositional differences among individuals’ dermal bacterial communities are at least partly driven by host genotypic variation as well as immunology (12).

10.1128/mBio.02945-19.4 FIG S3 Scatter plots of bacterial 16S composition in the dermal (ai) and epidermal (aii) compartments, and box plots of 16S OTU richness diabetic status (b), sex (c), and smoking habits (d). Download FIG S3, PDF file, 0.01 MB. Copyright © 2020 Bay et al. 2020 Bay et al. This content is distributed under the terms of the Creative Commons Attribution 4.0 International license.

10.1128/mBio.02945-19.5 FIG S4 NMDS plots of 16S communities of the epidermal (a, c, e, and g) and dermal (b, d, f, and h) communities. Color represents age (a and b), diabetic status (c and d), sex (e and f), and smoking habits (g and h). Download FIG S4, PDF file, 0.04 MB. Copyright © 2020 Bay et al. 2020 Bay et al. This content is distributed under the terms of the Creative Commons Attribution 4.0 International license.

The functional capabilities of the epidermal and dermal bacterial communities were computationally annotated to predict potential gene families. A PCA plot of the predicted genes demonstrates separate groupings of samples from the dermal and epidermal compartments (Fig. S6) based on predicted gene ortholog abundances. There was a significant difference (*P* < 1e−5) in relative gene abundances between the two compartments, suggesting a difference in overall functionality of the microbial communities. A heat map (Fig. 4a) and a simplified ridge plot (Fig. 4b) describing the 25 most significantly different pathways (all false discovery rates [*q* values] < 1e−5) clearly illustrate the variability in functionality throughout the stratified tissue. The difference in proportions of the pathways can lead to an inference of the functional relevance in the epidermal and dermal communities. The enriched pathways in the dermal community, such as nitrogen metabolism, bacterial chemotaxis, flagellar assembly, and lipopolysaccharide biosynthesis, indicate a possible dormant bacterial community that is initiating a biofilm mode of growth and survival by use of alternative energy pathways. Concurrently, the dermal depleted pathways include DNA replication, mismatch repair, homologous recombination, and pyrimidine/purine metabolism (Fig. 4), indicating bacteria with a decreased cell growth. These predictive, metagenomic analyses suggest a clear contrast in functional capability of the microbial communities between cutaneous compartments.

Various skin compartments exhibit specific milieus (2, 12). Hair follicles contain anoxic environments (12) that may allow anaerobic bacteria to thrive, cultivating their own unique localized microbiota (29). They stretch from the outermost layers of the epidermis deep into the dermis (14), contain a significant proportion of microorganisms, and should be considered pathways for the microbiota of the epidermis to enter the dermal layer of the skin and *vice versa*. Nevertheless, the dermal community is well protected against topical cutaneous antimicrobials, such as preoperational preparation antimicrobials that cannot penetrate the many cutaneous layers (30). The presence of a sebaceous plug in the infundibulum of hair follicles may furthermore hinder the penetration of drugs (31), and the deep regions of hair follicles thereby act as microbial reservoirs (32). This hidden dermal microbiota can easily reestablish the core of the epidermal microbiota or, during an operation, accidently be pushed further into the tissue and potentially initiate an infection.

In surgical site infections and chronic wounds, *Staphylococcus* spp. are ubiquitous (28, 33). Our findings of a medium abundance of *Staphylococcus* spp. in healthy skin (Fig. 2; Fig. S5b) suggest that this omnipresent genus possesses the potential to grow and outcompete other genera under favorable conditions. Coagulase-negative staphylococci (28), Cutibacterium acnes (34), and Pseudomonas aeruginosa (35) are also commonly found in surgical site infections and chronic wounds, as well as in healthy human skin (13). These potentially pathogenic species can cause complications when forced out of their natural habitat by surgical utensils or needles and possess the ability to develop chronic infections in opportunistic locations. These and other opportunistic pathogens sustain a low metabolism within the cutaneous compartments but thrive when favorable conditions exist.

Overall, our findings suggest an advanced adaptable epidermal microbiota and a less complex dermal core community in healthy human skin. Each of these communities can adapt to the compartment in which they thrive. Focusing on the dermal community may allow clinicians and researchers to simplify the relationship between the skin microbiome and skin disease, as it is less affected by environmental factors. These new findings may change the perception of the human skin microbiota as being entirely individual. However, distinctive details in the core microbiome may provide insight into health and genetics, as well as diet, lifestyle, and surroundings (16). While previous studies have attempted to map the entire epidermal skin microbiome (36), a greater understanding of the spatial scaling of microbial communities would also enhance our understanding of its function in chronic cutaneous diseases and infections. If the dermal communities serve as a reservoir for pathogenic bacteria that drive skin or soft tissue infections, strategies for preparing and managing patients undergoing surgery should be adjusted to reduce chances of complications. Speculatively, future preoperational procedures may include microbial analysis of the patient´s skin to predict potentially susceptible patients at risk for a postoperational infection. Correspondingly, the microbial composition of individuals suffering from chronic cutaneous conditions may predict upcoming flares of eczema and thereby improve prophylactic treatment. We therefore suggest that targeting different cutaneous compartments should be prioritized in future skin microbiome studies. A connection between autoimmune diseases or skin disorders and the microbiota may become clearer by investigating the dermal microbiota, as the dermal community is less affected by external factors and therefore possibly more stable. Further studies are needed to determine whether this core “universal microbiota” is present within dermal compartments across habitats. Nevertheless, future studies into chronic skin disorders would benefit from an increased focus on all skin compartments rather than just the epidermis, despite the invasive nature of biopsies.

The strength of this study is that the separation of cutaneous compartments allowed for the characterization of distinct communities at a level of detail not found in previous studies (2). The use of DNA metabarcoding allows for bacteria to be amplified even in very low concentrations and allows for noncultivable bacteria to also be described as part of the overall microbial composition (37). However, in future studies, a greater consideration of different skin habitats (including moist and sebaceous), ethnicities, and ages of participants should be included to extend the insight into the human microbiome. The addition of shotgun metagenomic and metatranscriptomic approaches would also considerably expand our understanding of the composition and functioning of the skin microbiome within different skin compartments (13).

### Conclusions.

Our results reveal similar microbial compositions between healthy hip and knee skin, while composition and functioning differed between the epidermal and dermal compartments. The dermal compartment generally contained a more homologous microbial composition among individuals, which was a specific subset of the epidermal microbiota. Together, these findings suggest that the human dermal microbiota is less variable than previously anticipated. This unexpected result is a major contribution to the understanding of human skin microbiota in health and disease, as the dermal communities may more accurately reflect the host’s genetic or immunological status rather than being a product of the host’s external environment.

## MATERIALS AND METHODS

### Ethical statement.

This study was approved by the Ethics Committee of the Capital Region (H-15012754) and by the data protection agency (HGH-2016-084) in Denmark. The study was conducted at the Orthopedic Department of Gentofte University Hospital, Copenhagen, Denmark, in November 2016.

### Participants.

Fifty participants were randomly selected by consecutive recruitment of patients eligible for primary knee arthroplasty. In Denmark, no formal records of patient ethnicities are recorded; however, participants reflected population demographics and were therefore primarily Caucasian. Patients signed a letter of informed consent after receiving oral and written information. Inclusion criteria were as follows: over 18 years of age and eligible for primary knee operation in the sampling period. Exclusion criteria were as follows: pregnancy, skin disorders, and active infections or use of antibiotics 4 weeks prior to sampling. The 50 participants enrolled included 24 males and 26 females with a mean age of 67 years (range, 47 to 85 years) and mean body mass index of 28 kg/m^2^ (range, 20 to 40 kg/m^2^). Three participants had diabetes. The group contained 7 active cigarette smokers, 14 former smokers, and 28 nonsmokers.

### Sample collection.

Skin biopsy specimens were collected by an orthopedic surgeon under aseptic conditions with laminar airflow in the operating room. Four punch biopsy specimens of 4 mm in diameter (Acu-Punch; Acuderm, Inc., Fort Lauderdale, FL, USA) were collected from nondisinfected skin of the opposite leg immediately prior to preparation for the primary knee arthroplasty. These samples included the following: two biopsy specimens from 2 cm proximal to the supero-lateral corner of the patella and two biopsy specimens from 2 cm proximal to the greater trochanter of the femur. One hip and one knee skin biopsy specimen from each patient were divided into superficial (1 mm) and remaining skin (3 mm) by scalpel in a modified version of the method described by Grice et al. (9). The separated biopsy specimens consisted of both (i) the epidermis and the superficial dermis, including the papillary dermal region (epidermal compartment), and (ii) the remaining dermis, including the reticular dermal region (dermal compartment) (see Fig. S1a and b in the supplemental material). Each part of the divided samples was transferred into RNA/DNA-free Eppendorf tubes (Eppendorf, Hamburg, Germany) before the addition of 1 ml RNAlater (Qiagen, Hilden, Germany). The samples were then stored at 5°C for up to 1 week and subsequently at –80°C.

10.1128/mBio.02945-19.3 FIG S1a Structure of skin biopsy specimens and bacterial aggregates in three dimensions. Confocal laser scanning microscopy image of a skin biopsy specimen illustrating the division into epidermal (1 mm) and dermal (3 mm) compartments, above and below the dashed line, respectively. The epidermal compartment contains the epidermis and the superficial dermis, including the papillary dermal region. The dermal compartment contains the remaining dermis, including the reticular dermal region. The microbiota of the stratum corneum and infundibulum of hair follicles are mainly included in the epidermal compartment, while the microbiota of the remaining hair follicles and glands are included in the dermal compartment. The white square indicates the zoomed image shown in Fig. S1b. Nucleated eukaryotes and bacteria (blue) are stained with DAPI (4′,6-diamidino-2-phenylindole), while erythrocytes (yellow) and surrounding tissue (green) appear due to autofluorescence. Download FIG S1a, TIF file, 0.8 MB. Copyright © 2020 Bay et al. 2020 Bay et al. This content is distributed under the terms of the Creative Commons Attribution 4.0 International license.

10.1128/mBio.02945-19.4 FIG S1b Healthy skin microbiota in the upper hair follicle are distributed in small bacterial aggregates and scattered single cells. Zoomed image of portion of Fig. S1a defined by a white square. Download FIG S1b, TIF file, 1.4 MB. Copyright © 2020 Bay et al. 2020 Bay et al. This content is distributed under the terms of the Creative Commons Attribution 4.0 International license.

### Sample preparation for Fig. S1a and b.

A second set of biopsy specimens, one hip and one knee biopsy specimen from each patient, were used for a parallel study (Lene Bay, Anne Brun Hesselvig, Anders Odgaard, and Thomas Bjarnsholt, unpublished data). These biopsy specimens were transferred into 9-ml S-Monovettes (Sarstedt, Nümbrecht, Germany) containing 4% formaldehyde and stored at 5°C for up to 1 week and subsequently embedded in paraffin. The preserved tissues were prepared as previously described (38) to obtain digital overview images of dry skin habitat sections (Fig. S1a and b).

### Sample preparation.

DNA extraction and PCR were performed in laminar flow hoods at the Centre for GeoGenetics, Copenhagen, Denmark. Tissues, along with extraction controls, were first lysed using a TissueLyser (Qiagen, Hilden, Germany) for 10 min at a frequency of 30 Hz. DNA was then extracted from the lysed skin samples using the MO BIO PowerViral environmental RNA/DNA isolation kit (Qiagen, Hilden, Germany) according to the manufacturer’s guidelines, and controls were treated the same way. Metabarcoding was performed on the bacterial V3-V4 16S region, using the 341F (5′-CCTAYGGGRBGCASCAG-3′) and reverse 806R (5′-GGACTACNNGGGTATCTAAT-3′) primers (39). Additionally, internal tags that ranged from 6 to 8 bp long were added to the primers to increase the number of samples multiplexed per library. The DNA content was measured using a Qubit double-stranded DNA (dsDNA) high-sensitivity assay kit (Invitrogen, Thermo Fisher Scientific, Waltham, MA, USA) and diluted to a concentration of 1 ng μl^−1^. PCR mixtures consisted of 1 μl of DNA extracts (sample or in-house positive control) or molecular biology-grade water (Thermo Fisher, Germany) for negative controls added to 0.2 μl of 5 U/μl AmpliTaq Gold (Applied Biosystems; Thermo Fisher Scientific, Waltham, MA, USA), 2.5 μl of 10× PCR Gold buffer, 2.5 μl of 25 mM MgCl_2_, 1 μl of each primer (at 25 mM/μl), 0.2 μl of 25 mM deoxynucleoside triphosphates (dNTPs) (Invitrogen, Thermo Fisher Scientific, Waltham, MA, USA), with cycling conditions consisting of 95°C for 5 min, 36 cycles of 95°C for 30 s, 56°C for 30 s, and 72°C for 30 s, and a final extension of 72°C for 10 min. Samples and controls were pooled into five pools and purified using a QiaQuick PCR purification kit (Qiagen, Hilden, Germany) per the manufacturer’s instructions. To prepare samples for sequencing, the TruSeq DNA PCR-free library preparation kit (Illumina, San Diego, CA, USA) was used per the manufacturer’s instructions, while excess adapters and primer dimers were removed by a final purification step using AMPure XP magnetic beads at a ratio of 1:1 volume of beads to PCR product (Beckman Coulter, Fisher Scientific, Hampton, NH, USA). DNA libraries were quantified on a Bioanalyzer (Agilent Technologies, Santa Clara, CA, USA) using the Agilent high-sensitivity DNA kit (Agilent Technologies, Santa Clara, CA, USA). The completed libraries were pooled at equimolar concentrations and sent for 250-bp paired-end sequencing on the Illumina MiSeq platform (Illumina, San Diego, CA, USA) at the National High-Throughput Sequencing Centre, Copenhagen, Denmark.

### Bioinformatic analysis.

Paired ends were merged and denoised (with a single nucleotide being the maximum permissible error) using the USEARCH (v.10.0) package. Sequencing libraries were demultiplexed using a custom script, which removed adapters, primers, and internal tags using CutAdapt (v.1.9.1) (40), while combined reads shorter than 400 bp were also removed. In order to limit erroneous assignment of reads to samples, reads were assigned only to samples when the unique combination of forward and reverse tags was found. Operational taxonomic units (OTUs) were clustered at the 97% similarity level in QIIME using the UCLUST algorithm (v.1.2.22) (41), before chimeras were detected and removed using the UCHIME *de novo* chimera checking algorithm (42). Taxonomy was assigned to OTU via Ribosomal Database Project (RDP) Classifier (43), using the SILVA (v.123) reference database. After quality filtering, the generated data set consisted of 3,477,595 high-quality 16S rRNA gene sequences with an average length of 420 bp (standard deviation [SD], ±20 bp). Finally, samples were rarefied to an equal sampling depth of 1,032 reads. This criterion yielded a total of 79 hip and 78 knee samples, spanning 74 dermal and 83 epidermal samples. In order to measure contamination within our samples, we included negative controls (with water replacing the DNA template) in every PCR setup (minimum of 1 negative with every 15 samples), while a further 6 extraction blanks were also processed and sequenced. Approximately half of these negatives had fewer than our minimum read threshold (1,032 reads) and were removed. Further investigation into the 12 negatives with sequencing suggested that there were a few common contaminants. Reads in the negatives were scattered in low abundance across 96 OTUs, but only 19 OTUs were present in more than 3 of these controls. These are listed in Table S2 as probable contaminants.

10.1128/mBio.02945-19.10 TABLE S2 Possibly contaminant bacterial OTU. In total, 23 negative controls were run (17 with water in PCRs and 6 extraction blanks), with 12 yielding our minimum of 1,032 reads. Persistence is the number of negative controls within which an OTU was found (out of 12). Download Table S2, DOCX file, 0.01 MB. Copyright © 2020 Bay et al. 2020 Bay et al. This content is distributed under the terms of the Creative Commons Attribution 4.0 International license.

### Statistical analysis.

All statistical analyses were performed within R (v.1.0.143), and all plots were generated within the ggplot2 package (44). Read counts were converted to relative abundances (the compositional percentage of an observed OTU relative to the total observations of OTUs in a sample) for downstream statistical analyses. Community similarities were calculated as Bray-Curtis similarity matrices (45) and visualized using nonmetric multidimensional scaling analyses. The effects of the skin compartment (epidermal or dermal compartment), anatomic location (knee or hip), and interpersonal variation on microbial composition were performed using the envfit function, all of which were performed using the vegan package (46). The effects of skin location and anatomic location were also tested on (log-normalized) operational taxonomic unit (OTU) richness (sum of all unique OTUs in a sample), with each serving as a fixed effect in their own model, while patient ID was included as a random effect. Mixed linear models were constructed using the lmer function, and significance was tested using the drop1 function to perform likelihood ratio tests (chi-square), using the lme4 package (47).

Due to substantial differences in community composition and OTU richness between the epidermal and dermal compartments in our combined analyses, samples were partitioned by their skin layer for further independent analyses. As before, Bray-Curtis similarity matrices were generated for each community and underwent NMDS analysis. Differences in bacterial composition between anatomic locations, biological sex, smoking, diabetic status, age, and interpersonal variation were assessed using the envfit function. Further analysis was performed using multivariate generalized linear models, which compared anatomic locations, biological sex, smoking, diabetic status, age, and patient ID against the bacterial community composition. Using a negative binomial distribution and 500 iterations, model term *P* values were calculated using the package MVABUND (48). Additionally, significant differences between the community similarity distributions (derived from Bray-Curtis similarity matrices) of the epidermal and dermal compartments were assessed using a Wilcoxon signed rank test. In testing effects on OTU richness, mixed modeling was performed to assess the effects of all explanatory variables (age, sex, smoking history, diabetic status, and anatomic location) on OTU richness (log normalized). As before, likelihood ratio tests (chi-square) were performed on the models to determine significance of explanatory variables.

The heat tree (Fig. 2) was generated using the Metacoder package within R (49). Initially, we calculated the abundance per taxon using the calc_taxon_abund function and the epidermal and dermal communities were compared using the compare_groups functions. The heat tree function was used to make phylogenetic trees, with the log_2_ (OTU richness_epidermal_/OTU richness_dermal_) ratio of median proportions plotted.

Indicator OTUs associated with the epidermal and dermal compartment communities were identified using indicator species analysis on all of the data combined. However, prior to analysis, OTUs occurring in five or fewer samples were removed. Indicator species were identified by determining statistical significance of species site-group associations using the signassoc function within the Indic species package (v.1.7.6) (50), while *P* values were corrected for multiple comparisons using Sidak’s correction (50–52). The taxonomy of each of these significantly different OTUs was further refined by performing a BLAST search against the NCBI 16S rRNA database and characterized by oxygen consumption based on published literature (Fig. 3).

PICRUSt was used to predict the functional profiles of organisms in each compartment using an extended ancestral state reconstruction algorithm to predict the presence of potential genes based on the 16S data (53). A principal-component analysis (PCA) was performed using the PICRUSt gene ortholog predictions. A permutational multivariate analysis of variance (PERMANOVA) test was performed using the R (54) software package vegan (55), with 99,999 permutations performed on the gene predictions to test for significant functional differences between the epidermal and dermal skin compartments. The genes were aggregated into pathways using the HUMAnN2 software package (56). Mixed linear regression models were fitted with the lme4 (57) package to the log-transformed Z-scores of relative pathway abundances, testing for significant enrichments of pathways between skin compartments, while patient ID and sample served as random effects. The false discovery rate was controlled using the Benjamini-Hochberg method (58) and expressed as *q* values. A heat map of the significantly differing pathways and a ridge plot showing the density distributions of intraindividual differences in Z-scores between compartments for the 25 most significant pathways were generated to illustrate these results. Data visualization was performed using the R packages ggplot2 (44), ggridges (59), and pheatmap (60).

### Data availability.

All sequencing data were uploaded to the NCBI Sequence Read Archive under BioProject accession no. PRJNA510725.
