# Supplementary figures and images for: Universal Dermal Microbiome in Human Skin
Source: mBio. 2020 Feb 11;11(1):e02945-19. doi: 10.1128/mBio.02945-19 (PMC7018652; doi:10.1128/mBio.02945-19)

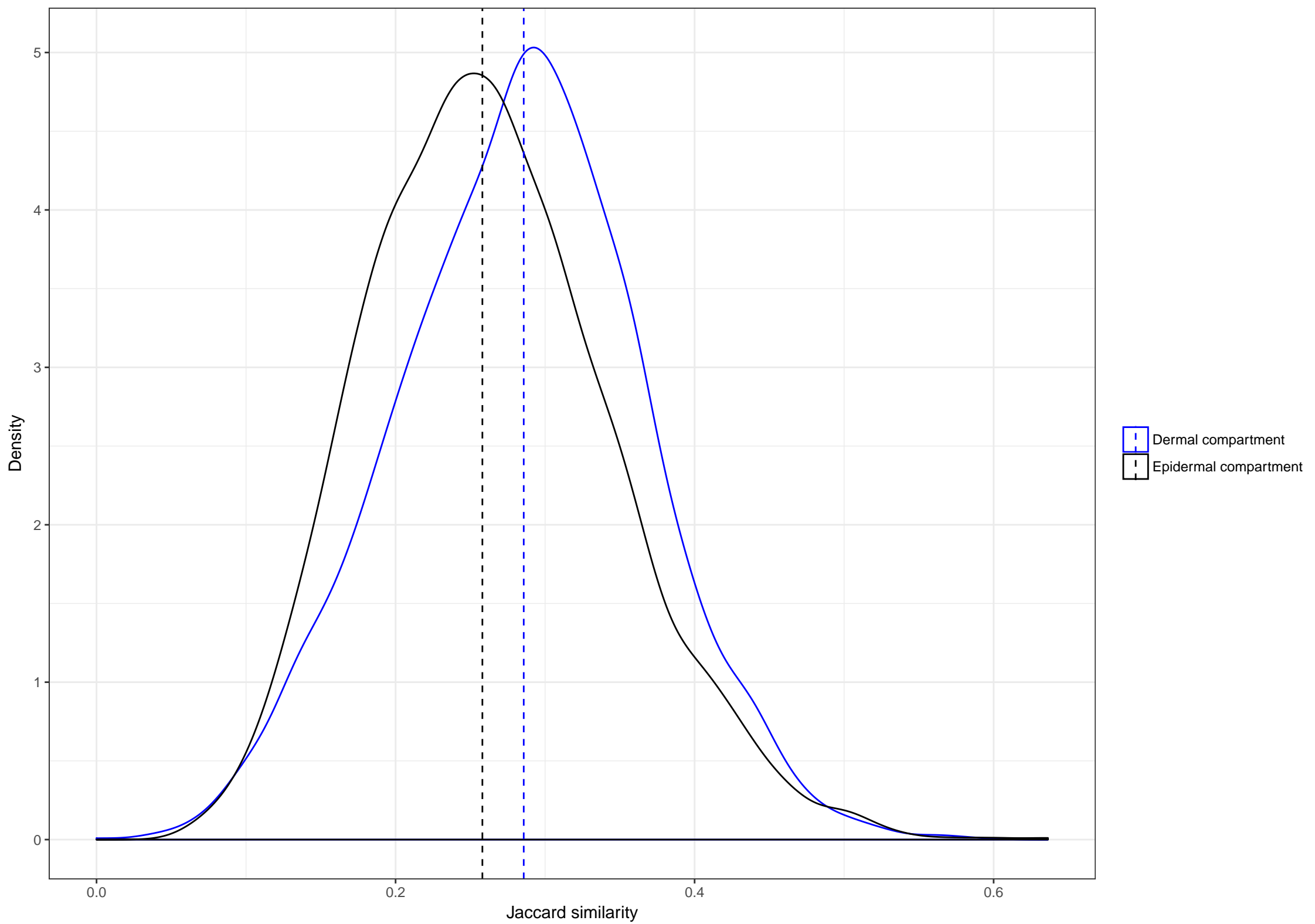

Supplement: FIG S2 [file mBio.02945-19-sf002.pdf]

a)

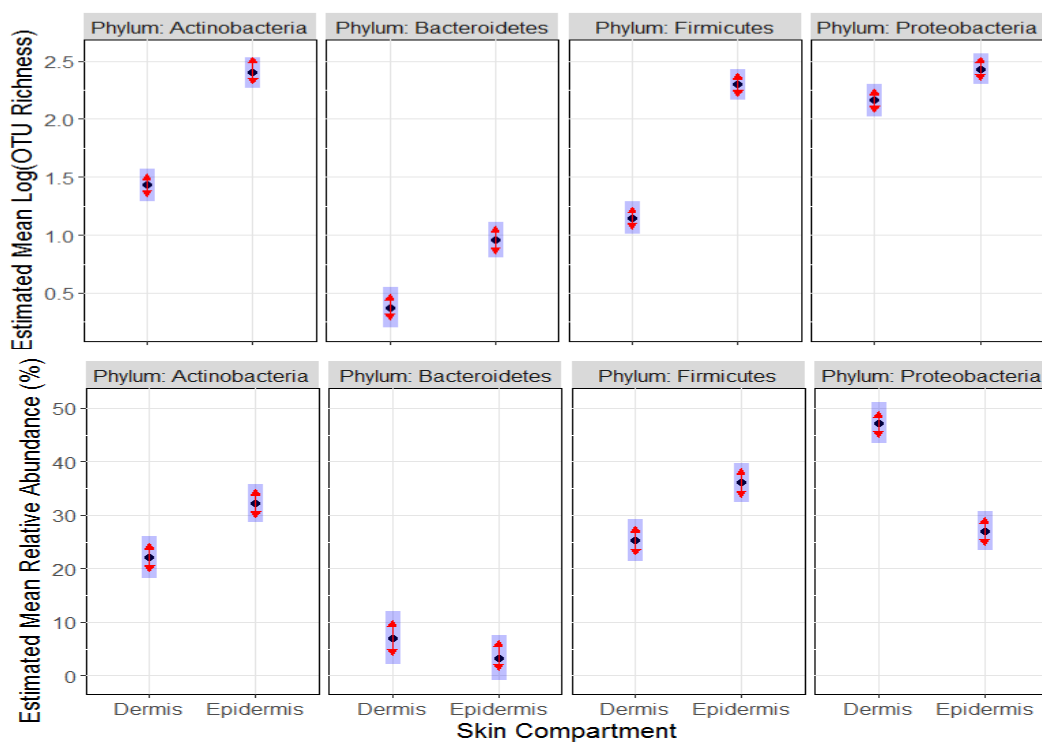

b)

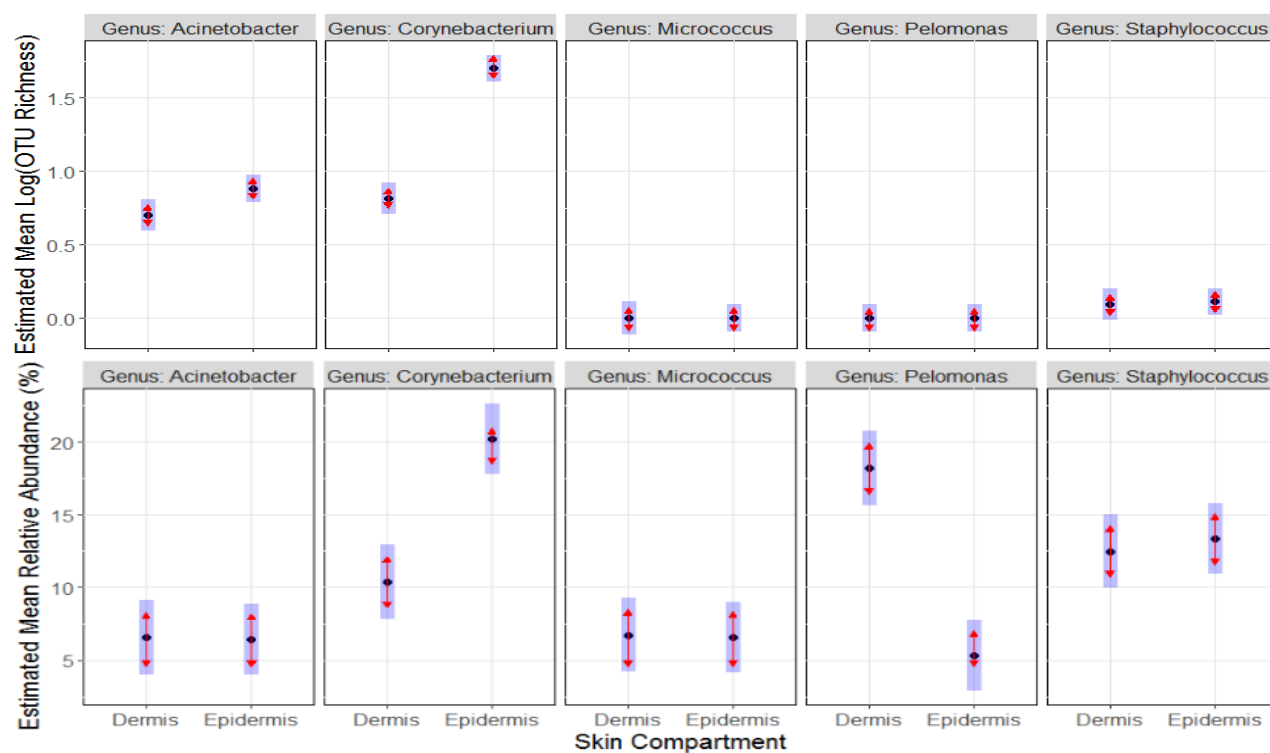

**Supplementary Figure 5: Box plots of top four phyla and top five genera.**

Supplement: FIG S5 [file mBio.02945-19-sf005.pdf]

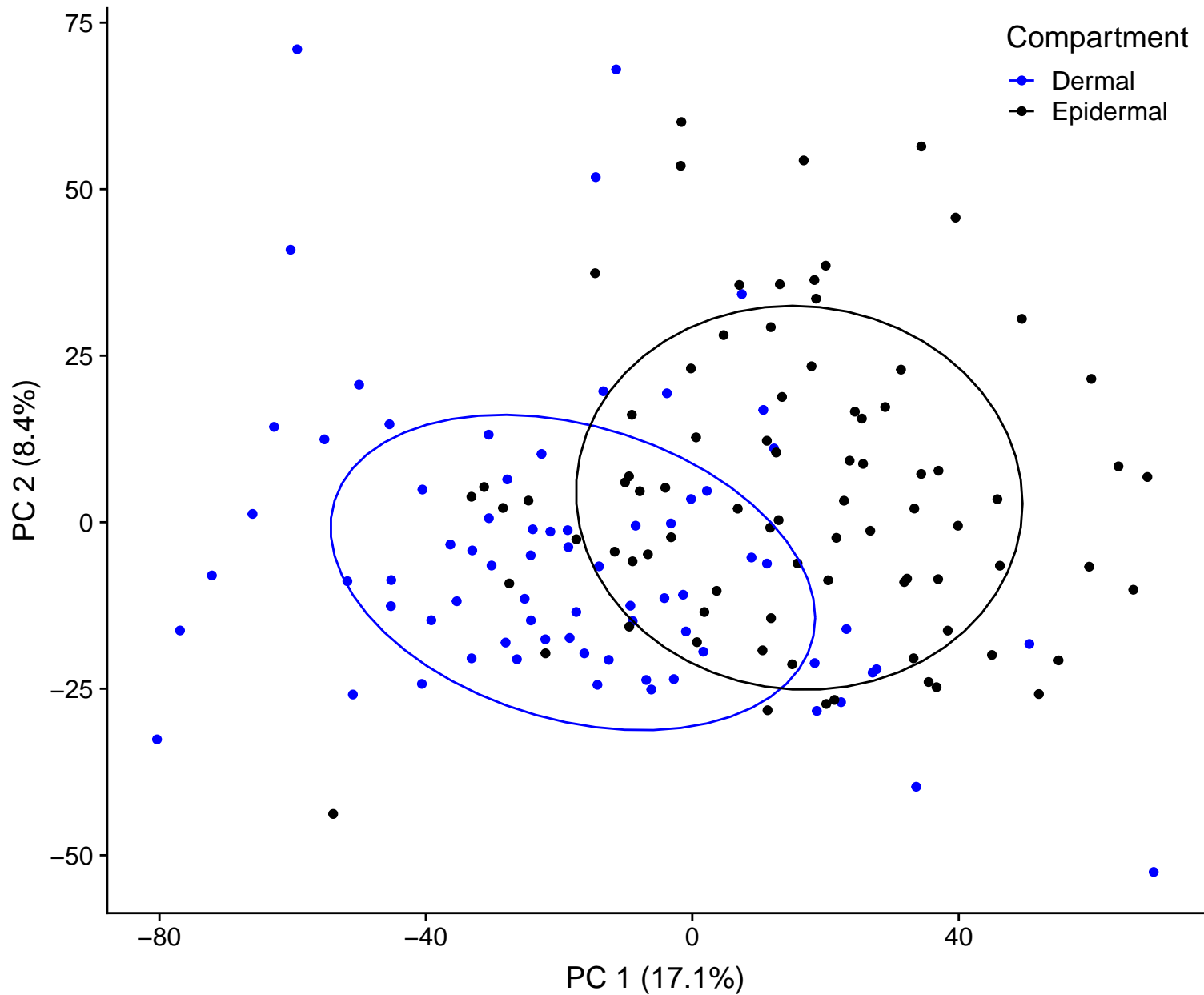

Supplement: FIG S6 [file mBio.02945-19-sf006.pdf]

Pathways

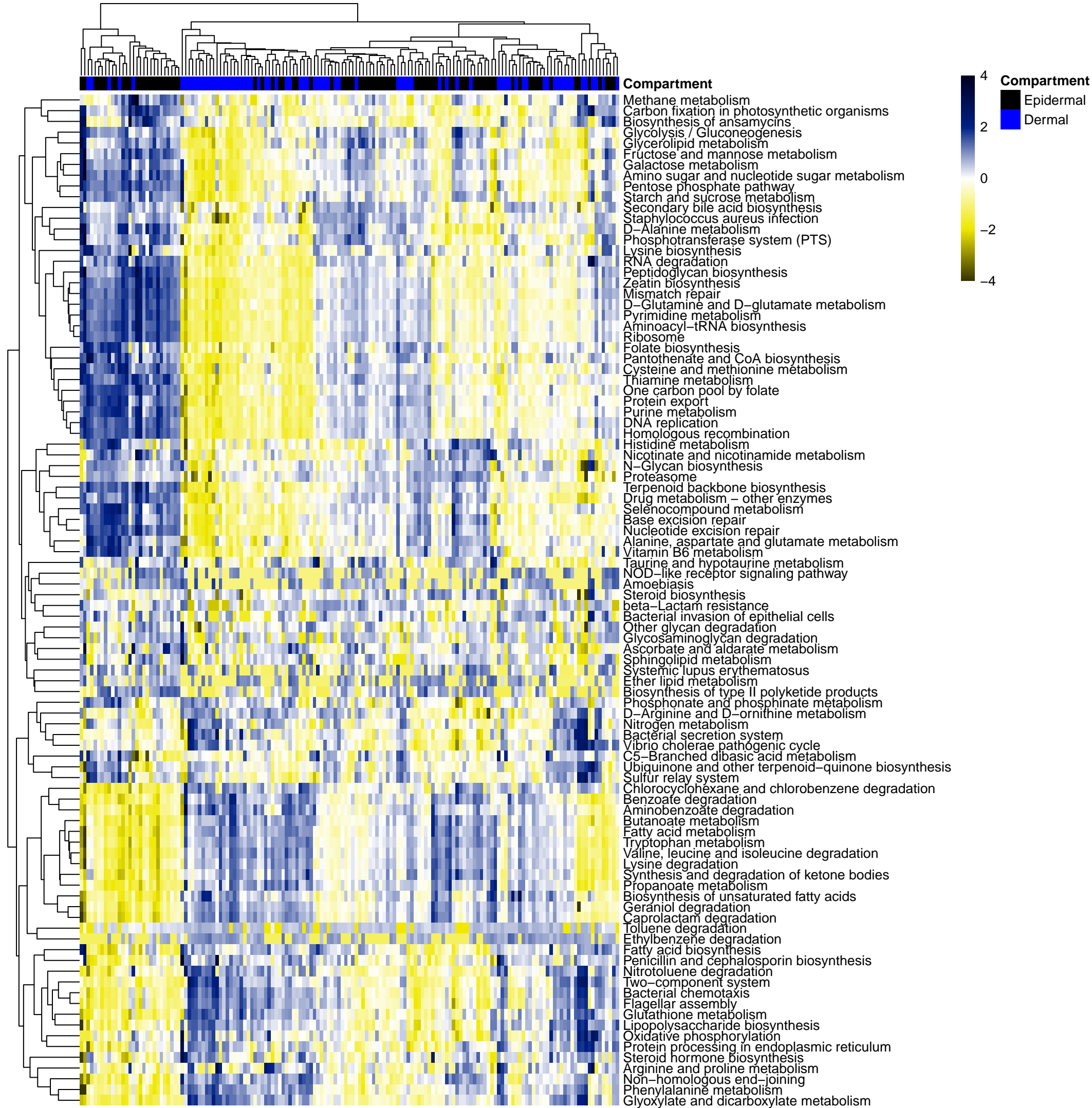

155 Samples

Supplement: FIG S7 [file mBio.02945-19-sf007.pdf]

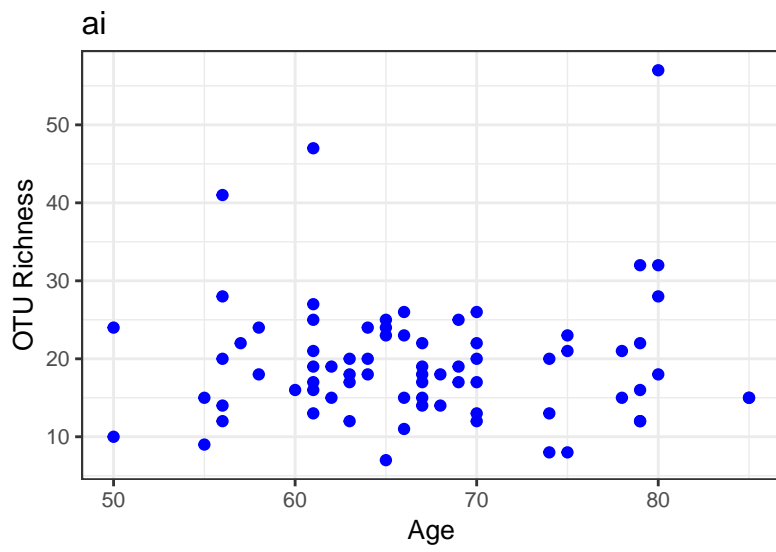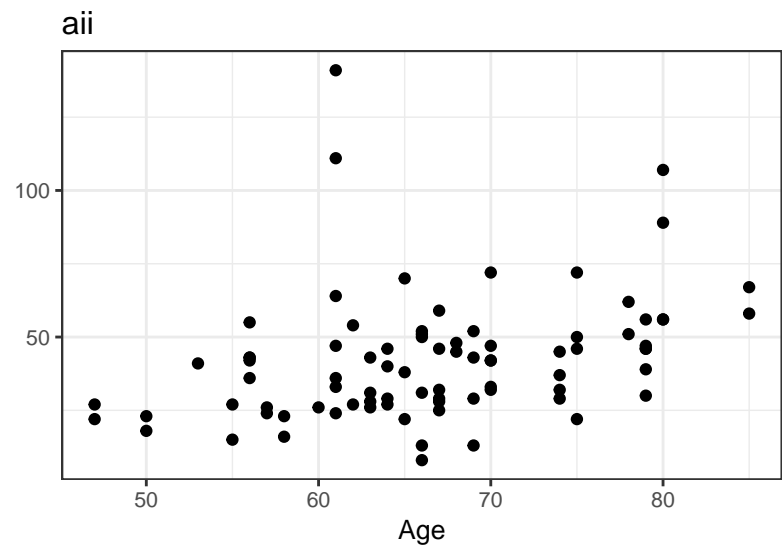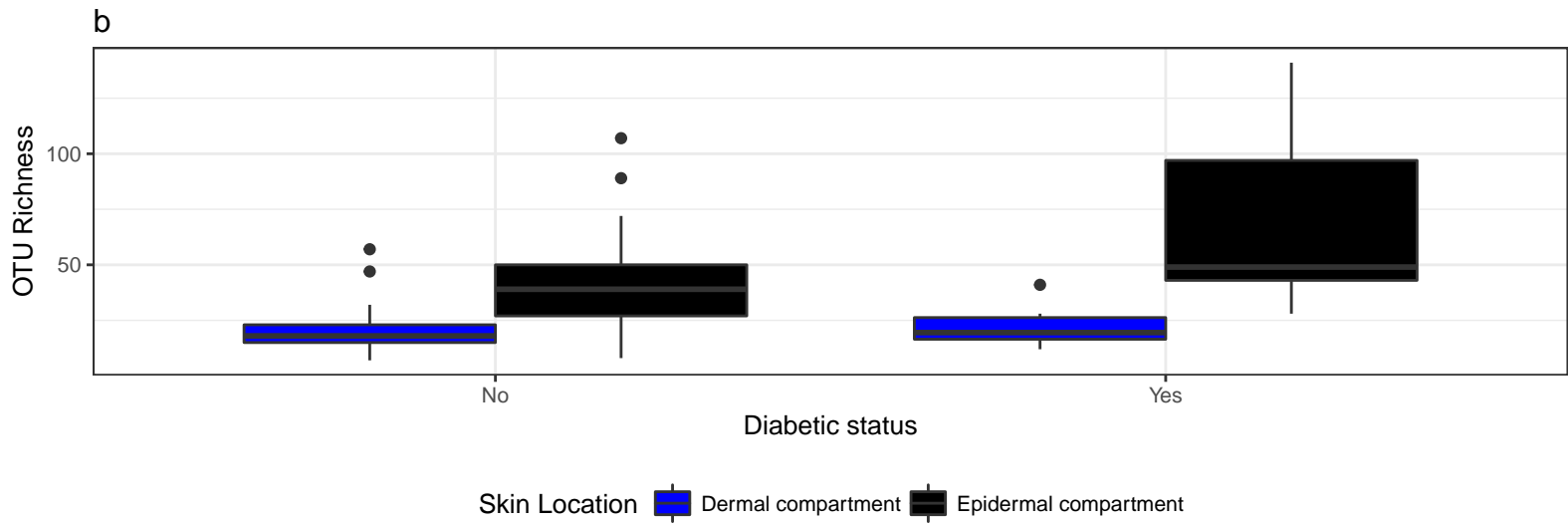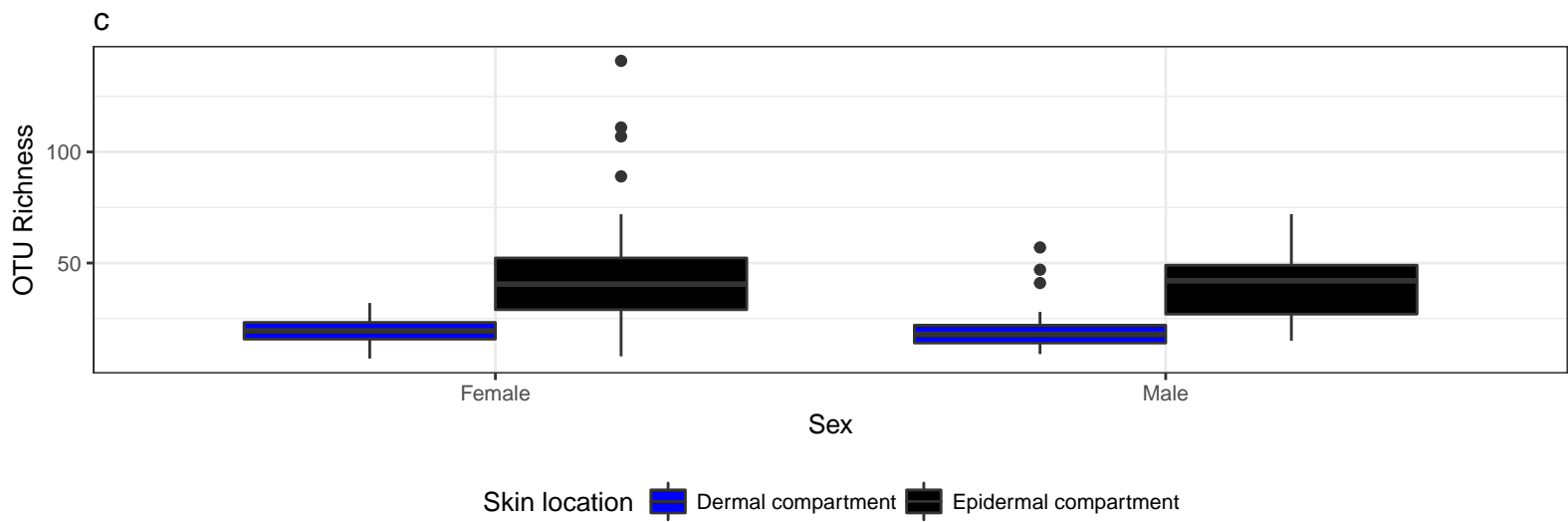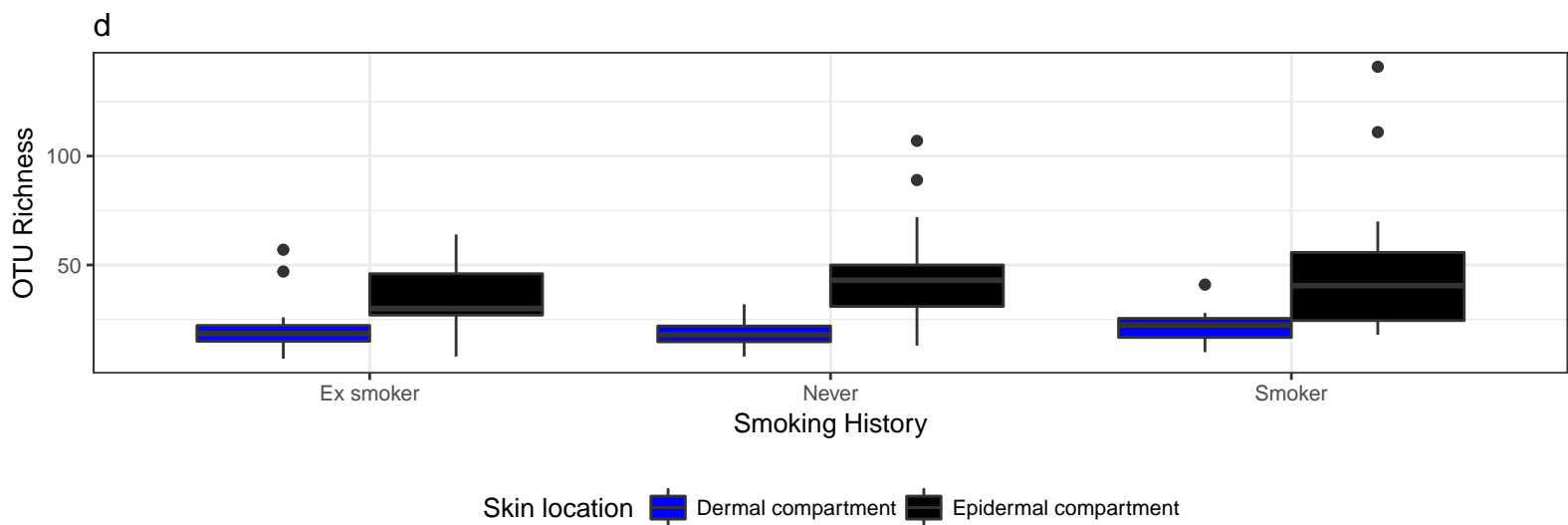

Supplement: FIG S3 [file mBio.02945-19-sf003.pdf]

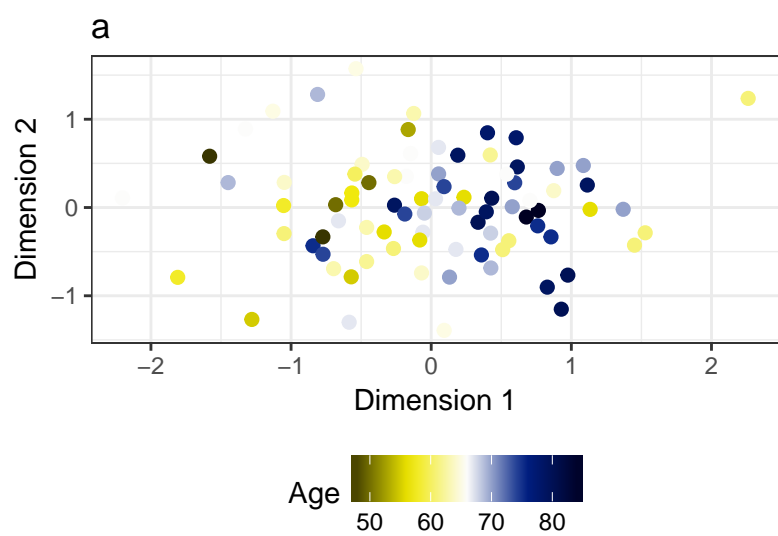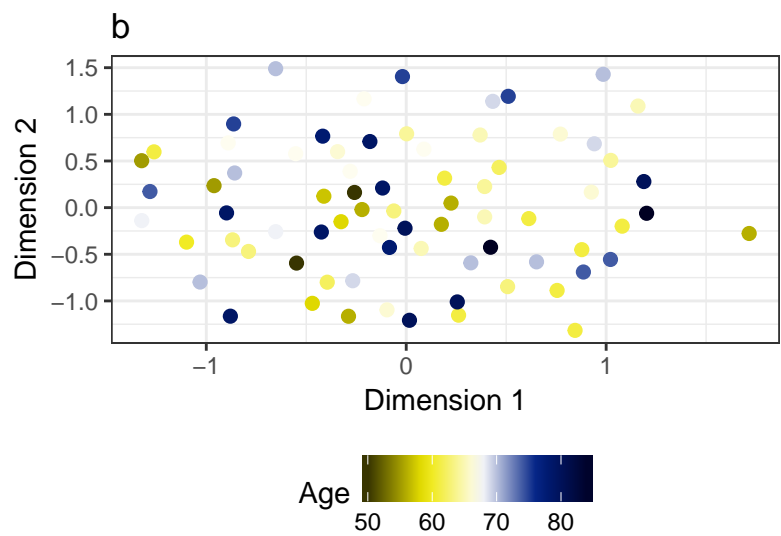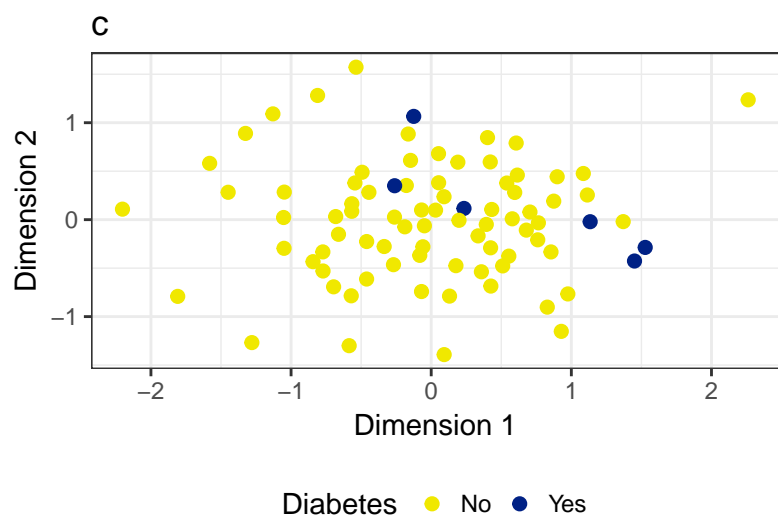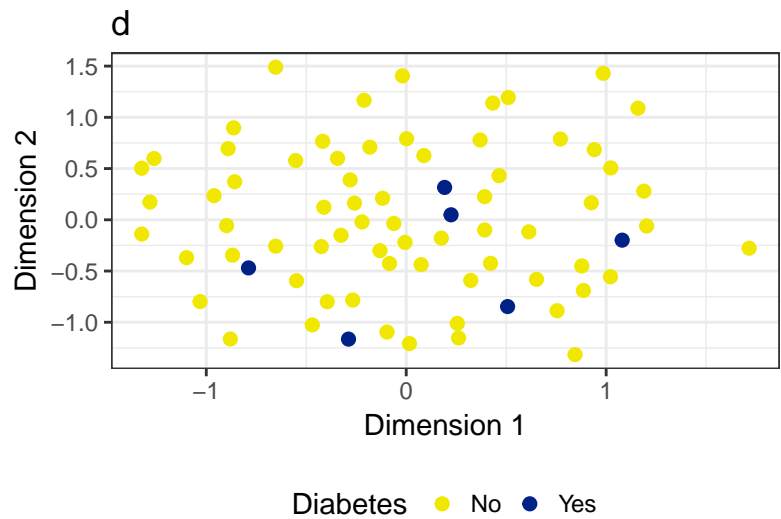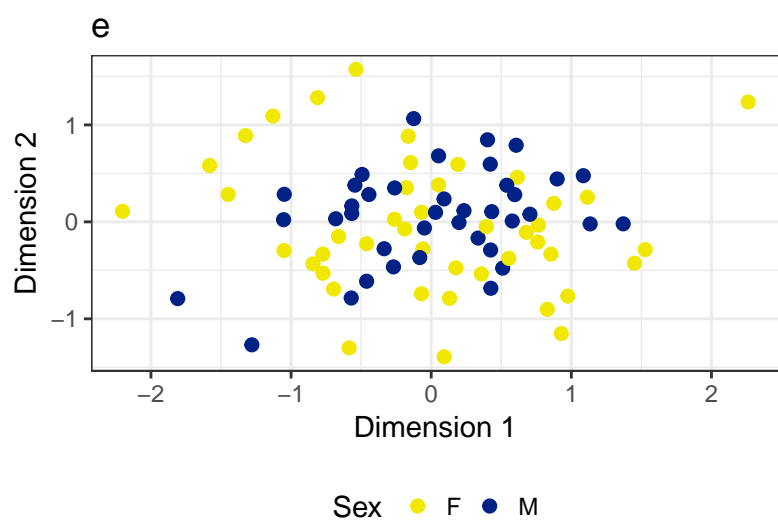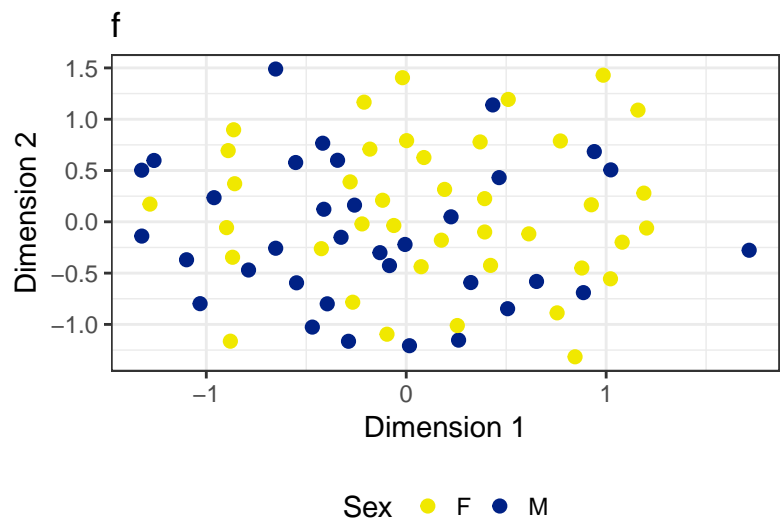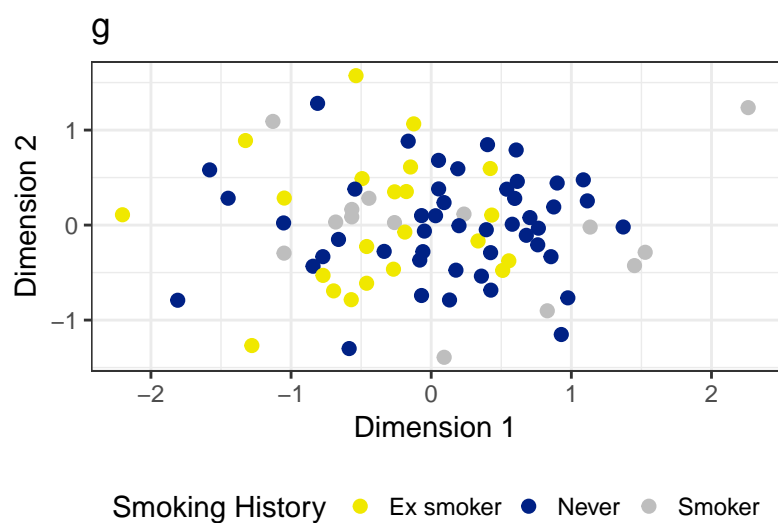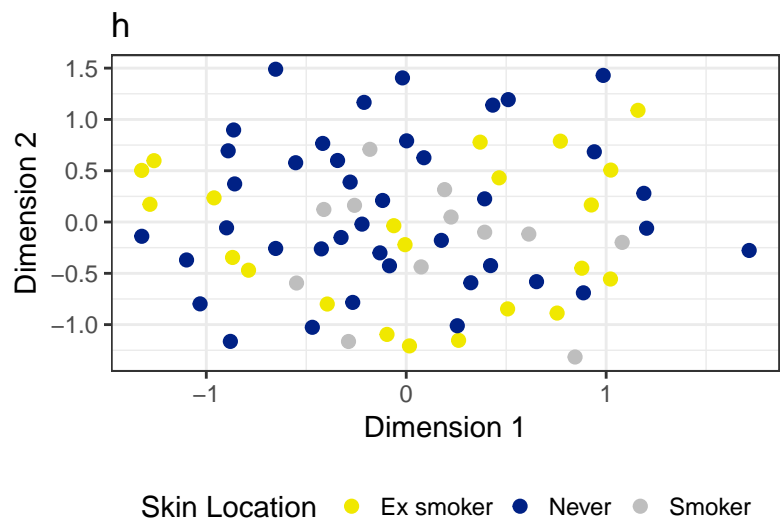

Supplement: FIG S4 [file mBio.02945-19-sf004.pdf]

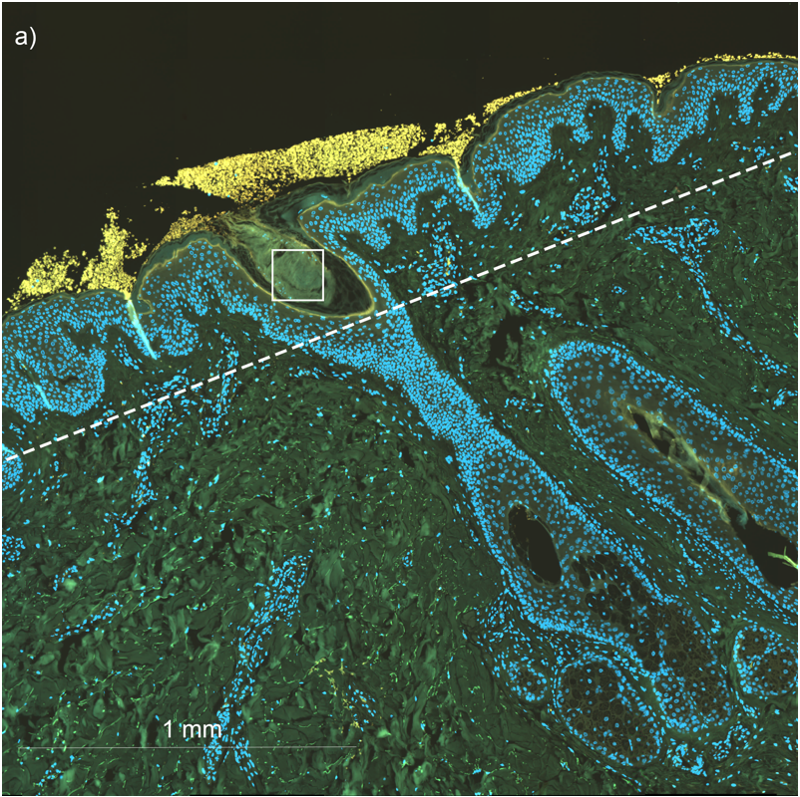

Supplement: FIG S1a [file mBio.02945-19-sf01a.tif]

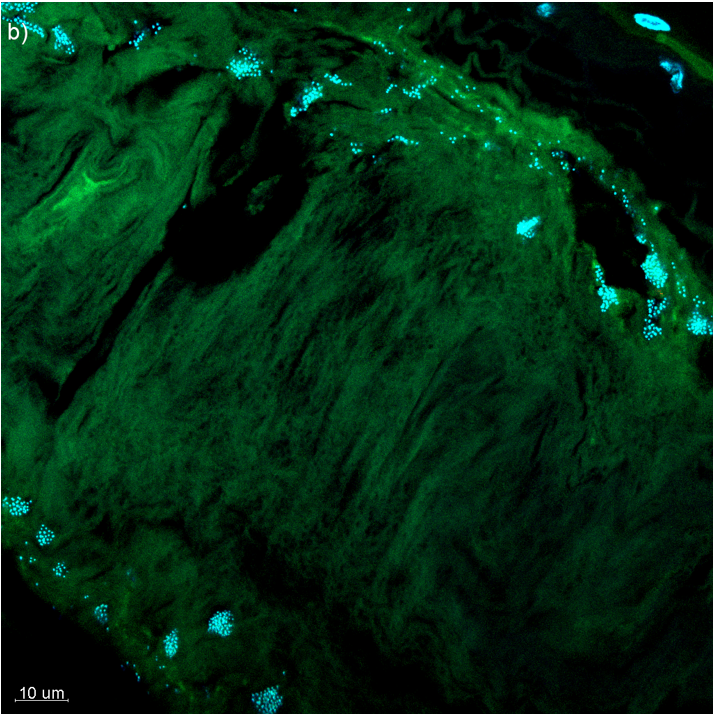

Supplement: FIG S1b [file mBio.02945-19-sf01b.tif]
