# Supplementary material for: Universal Dermal Microbiome in Human Skin
Source: mBio. 2020 Feb 11;11(1):e02945-19. doi: 10.1128/mBio.02945-19 (PMC7018652; doi:10.1128/mBio.02945-19)
Supplement: TABLE S1 [file mBio.02945-19-st001.docx]

|  | **All Data** | | |
| --- | --- | --- | --- |
|  | **Mixed linear modeling**  *(Chisq, p-value)* | **Envfit function**  *(R^2^, p-value)* | **Combined model w/ drop1 function** *(LTR, p-value)* |
| **Skin Compartment** | 95.817, **< 2.2e^-16^** | 2.329, **0.001** | 95.961, **<2e^-16^** |
| **Anatomic Location** | 0.447, 0.5037 | 0.005, 0.426 | 0.591, 0.442 |
|  | **Epidermal Compartment** | | |
|  | **Mixed linear modeling**  *(LTR, p-value)* | **Envfit function**  *(R^2^, p-value)* | **Multivariate general linear modeling** *(p-value)* |
| **Anatomic Location** | 3.220, 0.073 | 0.006, 0.623 | **0.046** |
| **Interpersonel variation** | n/a | 0.155, **0.001** | **0.002** |
| **Age** | 10.998, **0.001** | 0.160, **0.002** | **0.006** |
| **Sex** | 0.173, 0.895 | 0.007, 0.570 | **0.020** |
| **Smoking habits** | 3.431, 0.180 | 0.079, **0.011** | **0.014** |
| **Diabetes status** | 7.294, **0.007** | 0.034, 0.062 | **0.036** |
|  | **Dermal Compartment** | | |
|  | **Mixed linear modeling**  *(LTR, p-value)* | **Envfit function**  *(R^2^, p-value)* | **Multivariate general linear modeling** *(p-value)* |
| **Anatomic Location** | 0.071, 0.789 | 0.008, 0.538 | 0.138 |
| **Interpersonel variation** | n/a | 0.035, 0.276 | **0.004** |
| **Age** | 0.803, 0.370 | 0.003, 0.870 | **0.010** |
| **Sex** | 0.020, 0.887 | 0.025, 0.181 | 0.094 |
| **Smoking habits** | 1.699, 0.428 | 0.019, 0.606 | 0.142 |
| **Diabetes status** | 0.209, 0.647 | 0.018, 0.265 | 0.363 |

**Supplementary Table 1: Overview of the statistics**.
