# Supplementary material for: Universal Dermal Microbiome in Human Skin
Source: mBio. 2020 Feb 11;11(1):e02945-19. doi: 10.1128/mBio.02945-19 (PMC7018652; doi:10.1128/mBio.02945-19)
Supplement: TABLE S2 [file mBio.02945-19-st002.docx]

| **Possibly contaminant bacterial OTU** | | | |
| --- | --- | --- | --- |
| **Taxonomy** | **Persistence** | **Taxonomy** | **Persistence** |
| *Atopobium* sp. | 12 | *Ruminococcaceae sp.* | 5 |
| *Cellulomonas* sp. | 8 | *Frankiales sp.* | 4 |
| *Conchiformibius* sp. | 8 | *Porphyromonas* sp. | 4 |
| *Corynebacterium* sp. | 7 | *Stenotrophomonas* sp. | 4 |
| *Prevotella* sp. | 7 | *Moheibacter* sp. | 4 |
| Lachnospiraceae sp. | 6 | Lachnospiraceae sp. | 4 |
| *Lactococcus* sp. | 5 | *Actinomyces* sp. | 4 |
| *Anaerococcus* sp. | 5 | *Prevotella sp.* | 4 |
| *Luteimonas* sp. | 5 | *Hymenobacter sp.* | 4 |
| *Moraxella* sp. | 5 |  |  |

**Supplementary table 2: List of possibly contaminant bacterial OTU.**
